# Pursuing Precision Medicine in Managing Rheumatoid Arthritis

**DOI:** 10.1111/1756-185X.70239

**Published:** 2025-04-23

**Authors:** Satoshi Kubo, Yoshiya Tanaka

**Affiliations:** ^1^ Department of Molecular Targeted Therapeutics University of Occupational and Environmental Health Kitakyushu Japan; ^2^ The First Department of Internal Medicine University of Occupational and Environmental Health Kitakyushu Japan

## Abstract

Rheumatoid arthritis, characterized by immune dysregulation and joint destruction, is managed through a stepwise algorithm that combines methotrexate with biological and targeted synthetic disease‐modifying antirheumatic drugs. Despite considerable advances, the lack of reliable biomarkers for selecting the most effective medication, especially in Phase II and beyond, remains a significant obstacle. As a result, achieving early clinical remission in all patients continues to be challenging. Rheumatoid arthritis demonstrates considerable clinical and molecular diversity, influenced by both genetic predispositions and environmental factors. Recent scientific and technological advances have shed light on the pathogenesis of rheumatoid arthritis, facilitating the stratification of patients into distinct phenotypic subgroups and potentially optimizing the choice of targeted therapies. However, persistent challenges include the high costs and logistical demands of these methodologies, as well as the complexities of conducting large‐scale clinical trials. This review highlights the intricate pathogenesis of rheumatoid arthritis and underscores the need to address the disease's heterogeneity through precision medicine. Moving forward, a deeper investigation into rheumatoid arthritis pathogenesis, encompassing both genetic and environmental factors, is crucial. Pursuing precision medicine, grounded in accurate patient stratification, should be embraced as a “moonshot” objective in rheumatoid arthritis treatment, aiming to achieve transformative breakthroughs in management.

## 1 Introduction

Precision medicine represents an aspirational target in contemporary medical practice for numerous diseases [1, 2]. In the United States, a national initiative is advancing precision medicine as the foundational strategy for treatment, employing molecular targeted therapeutics for malignant tumors. For instance, in the case of non‐small cell lung cancer, substantial response rates have been realized by identifying oncogenic mutations including EGFR, ALK, ROS1, BRAF, and RET and administering targeted therapies specifically designed to counteract these genetic anomalies [3, 4].

The realization of precision medicine constitutes a paramount concern in the treatment of autoimmune diseases [1]. Defined as “optimal patient stratification to maximize therapeutic efficacy,” precision medicine becomes practicable through meticulous stratification, enabling the selection of the most effective treatment [5]. Such optimal stratification entails a detailed elucidation of the diverse pathogenic mechanisms unique to each patient and the subsequent grouping of patients with analogous pathological profiles. Generally, the suppression of signals pivotal to the disease state enhances the efficacy of a drug, whereas the suppression of nonspecific signals heightens the risk of adverse events. Therefore, the fundamental principle of precision medicine is to selectively inhibit only those signals essential for managing the disease state, avoiding the suppression of extraneous signals. Consequently, the critical challenge lies in detecting the variability among patients. The advancement of immunology, primarily through mouse models, has significantly influenced the treatment of autoimmune diseases. Nonetheless, the diversity of pathological mechanisms within each patient is frequently underestimated [6].

Presently, single‐cell transcriptomics analysis yields high‐resolution data, enabling the ex vivo evaluation of patient specimens [7, 8]. This sophisticated analytical approach has identified previously unrecognized cell types involved in the pathogenesis of rheumatoid arthritis, enhancing our understanding of its complex biological underpinnings [9, 10]. However, the substantial cost associated with single‐cell transcriptomics analysis necessitates a significant financial investment to manage the volume of cases required for effective subgrouping. Furthermore, precision medicine studies demand an even greater number of cases. In fact, a precision medicine approach for immune disorders has not yet been firmly established [6, 11, 12]. Rheumatoid arthritis, one of the most prevalent autoimmune diseases, affects 23 million people globally. It leads to joint destruction driven by immune system dysregulation and abnormal bone metabolism, resulting in functional impairment and an increased mortality rate [13, 14, 15]. Therefore, the primary objective of treatment for rheumatoid arthritis is to inhibit joint destruction. Strategies to achieve this goal have been delineated in the European Alliance of Associations for Rheumatology (EULAR) treatment recommendations [16, 17]. This treatment strategy is structured in multiple phases. At the initial diagnosis of rheumatoid arthritis (Phase I), treatment begins with methotrexate or conventional synthetic DMARDs (csDMARDs). If methotrexate proves ineffective, molecular targeted therapy is introduced (Phase II). Should this targeted therapy fail to achieve the desired outcomes, another molecular targeted therapy is substituted (Phase III). In these treatment algorithms, a significant challenge is the absence of appropriate biomarkers for selecting molecular targeted therapies in Phase II and Phase III. Consequently, approximately 40% of patients do not respond adequately to treatment in Phase II, leading to prolonged use of costly molecular targeted therapies with limited efficacy. Therefore, the ability to select highly effective drugs from the outset is identified as a crucial clinical issue [16, 18].

**FIGURE 1:**
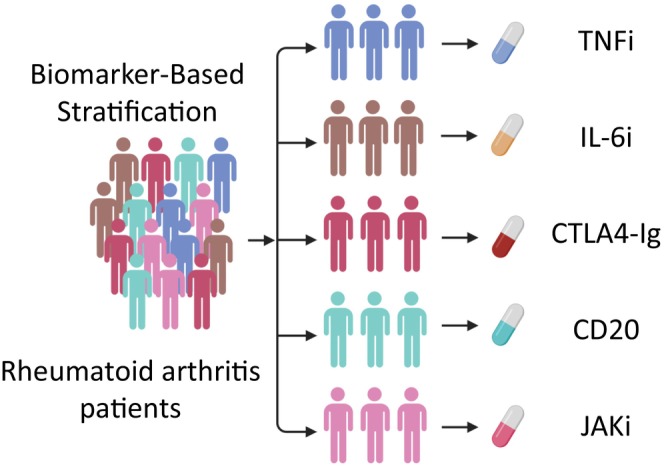
Biomarker‐based stratification and tailored treatment selection in medical practice. By stratifying patients based on appropriate biomarkers that accurately reflect their pathogenesis, it becomes feasible to tailor therapy using different classes of molecular targeted therapies. This approach enhances the precision of treatment strategies. IL‐6Ri, anti‐IL‐6 receptor antibodies; JAKi, JAK inhibitors; TNFi, TNF inhibitors.

Currently, five classes of molecular targeted therapies are available for rheumatoid arthritis, each focused on specific biological targets: the cytokines TNF (tumor necrosis factor) and IL‐6 (interleukin‐6), mechanisms of T‐cell activation, CD19 positive B cells, and the tyrosine kinase JAK (Janus kinase). These therapies are designed to specifically disrupt key inflammatory pathways involved in rheumatoid arthritis. Selecting molecular targeted therapies tailored specifically to an individual patient's pathogenesis is crucial. Therefore, identifying reliable biomarkers is essential for achieving this goal (Figure 1).

## 2 Diversity of Rheumatoid Arthritis Patients

Patients with rheumatoid arthritis demonstrate heterogeneity, manifested through clinical and immunological diversity. Clinically, these patients can be classified based on the presence or absence of biomarkers, such as rheumatoid factor or anti‐CCP antibodies. Those testing positive for these markers generally have a higher risk of progressive joint destruction. Moreover, clinical manifestations vary, with some patients developing extra‐articular complications like interstitial lung disease or vasculitis, while others remain unaffected by these conditions. Additionally, the prevalence of rheumatoid arthritis differs across age groups, resulting in distinct disease patterns and varying responses to therapy between younger and older patients.

Autoimmune diseases such as rheumatoid arthritis are shaped by both genetic predispositions and environmental factors [19]. On the genetic front, numerous single nucleotide polymorphisms (SNPs) have been recognized as risk factors for rheumatoid arthritis, with more than 120 SNPs identified in the latest genome‐wide association studies (GWAS) [20]. The presence or absence of these SNPs influences susceptibility, disease severity, and the efficacy of treatment responses. Notably, HLA‐DRB1, with its high reproducibility, is considered a genetic predisposition for rheumatoid arthritis across different ethnicities [21, 22]. Specifically, the amino acid sequence from positions 70 to 74 within the HLA beta chain, known as the shared epitope, plays a crucial role in the development of the disease. Given HLA's critical role in presenting antigenic peptides to T cells, these polymorphisms are believed to contribute to T cell activation, which is a key factor in the pathogenesis of rheumatoid arthritis. Another significant genetic predisposition for rheumatoid arthritis, apart from HLA, involves the T cell signaling regulatory molecule, protein tyrosine phosphatase non‐receptor type 22 (PTPN22) [23]. It is hypothesized that the interaction between PTPN22 and peptidylarginine deiminase enzymes leads to an exaggerated immune response to citrullinated proteins. Epigenetic modifications of the synovial tissue also play a crucial role in the diversity observed among rheumatoid arthritis patients. DNA methylation, a typical form of such modifications, contributes to the variability in disease expression and progression [24]. This epigenetic alteration can affect gene expression involved in inflammation and joint destruction, further complicating the clinical picture of rheumatoid arthritis. Environmental factors also play a crucial role in the heterogeneity of rheumatoid arthritis. For instance, smokers are at a higher risk of developing the disease compared to nonsmokers and typically exhibit less favorable responses to treatment. Other factors, such as periodontal disease, obesity, levels of physical activity, and dietary habits, further influence disease activity and overall patient health [25, 26, 27]. Thus, it is insufficient to account for patient diversity in rheumatoid arthritis solely on the basis of genetic predisposition. In addition, although they are not directly involved in the pathogenesis of rheumatoid arthritis, broader socioeconomic conditions, including national and individual financial resources, can affect opportunities for early diagnosis and treatment. This disparity in healthcare access contributes to variability in disease outcomes. Similarly, educational levels impact health literacy, influencing patients' understanding of their condition and their ability to seek and adhere to appropriate treatment strategies.

**FIGURE 2:**
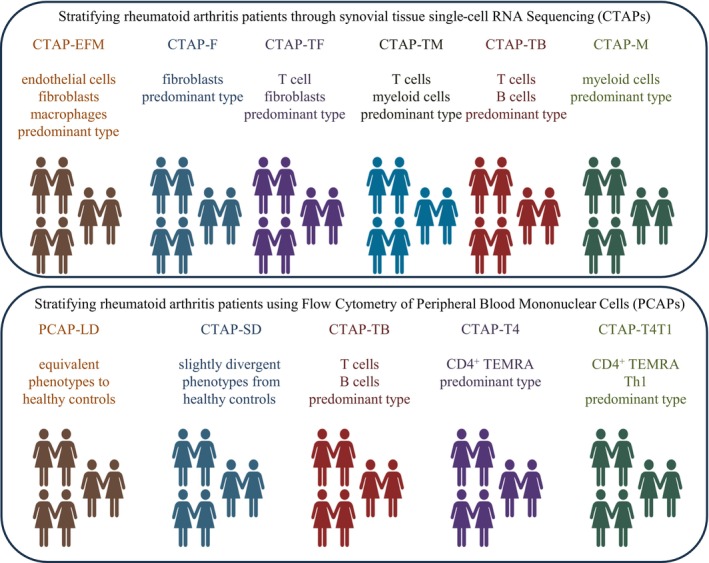
Two studies aimed to stratify rheumatoid arthritis patients using different biological markers. The upper figure illustrates the subdivision of synovial membrane samples from rheumatoid arthritis patients into distinct subpopulations, termed cell‐type abundance phenotypes (CTAPs), using single‐cell RNA sequencing. The lower figure depicts stratification based on peripheral blood samples from rheumatoid arthritis patients, classified into distinct populations termed peripheral blood cell abundance phenotypes (PCAPs), utilizing flow cytometry analysis.

The considerable diversity among rheumatoid arthritis patients, driven by genetic, environmental, socioeconomic, and educational factors, poses substantial challenges to achieving precision medicine. The quest for precision medicine in rheumatoid arthritis has therefore been a focal point of research efforts [28]. International consortiums have conducted rigorous studies on the synovial tissues of patients using single‐cell transcriptomics analysis, leading to the identification of new subsets of synovial fibroblasts [9, 29]. Additionally, a significant study involving 70 patients helped delineate the disease's heterogeneity [30]. Researchers analyzed over 314 000 synovial cells, using hierarchical clustering to identify six distinct Cell‐Type Abundance Phenotypes (CTAPs) based on cell‐type frequency (Figure 2). The CTAP‐EFM phenotype predominantly features endothelial cells, fibroblasts, and myeloid cells. CTAP‐F is rich in fibroblasts; CTAP‐TF, in T cells and fibroblasts; CTAP‐TB, in both T and B cells; CTAP‐TM, in T cells and myeloid cells; and CTAP‐M, mainly in myeloid cells. Further analysis reveals that CTAP‐TB is enriched with follicular and peripheral T cells, indicating a strong adaptive immune response. CTAP‐TF contains numerous cytotoxic T cells and CXCL12‐positive fibroblasts, suggesting involvement in immune recruitment and inflammation, whereas CTAP‐M is characterized by a high presence of inflammatory myeloid cells. These findings indicate that the traditional cell populations considered significant in rheumatoid arthritis do not manifest uniformly across all cases, suggesting that molecular targeted therapies may need customization to effectively address the specific cellular environments in individual patients. However, the study did not address whether these subpopulations respond differently to existing molecular targeted therapies.

## 3 A Phase IV Study Targeting Precision Medicine Through the Use of IL‐6 Inhibitor

The most distinguished research in the field of precision medicine for rheumatoid arthritis is exemplified by the R4RA (rituximab vs. tocilizumab in anti‐TNF inadequate responder patients with rheumatoid arthritis) study, a clinical trial underpinned by synovial biopsy [31, 32]. The R4RA study, a Phase IV clinical trial, focused on patients with rheumatoid arthritis who had previously demonstrated an inadequate response to TNF inhibitors. Utilizing biopsy samples from the synovial tissue, patients were classified into two groups: a B cell‐rich group, characterized by high expression of B cell‐related genes, and a B cell‐poor group, marked by low expression of these genes. The trial then employed a randomized controlled methodology to evaluate the efficacy of rituximab and tocilizumab within each stratified group. When patients were segregated into two groups based on transcriptome analysis, the response rates measured by CDAI revealed that only 36% of the B cell‐poor group responded to rituximab, whereas tocilizumab achieved a higher response rate of 63% in the same group. When comparing the gene expression profiles of synovial tissue to the response rates, it was observed that rituximab elicited a high response rate in cases exhibiting increased expression of immunoglobulin‐related and lymphocyte‐related genes. Conversely, tocilizumab showed heightened efficacy in cases where there was an upregulation of macrophage‐related genes and genes associated with metabolic pathways. This study demonstrated that stratifying patients using next‐generation sequencing analysis, rather than traditional pathological immunostaining, allows for the selection of appropriate treatment drugs. This approach has raised substantial expectations for the advancement of precision medicine.

Building on these findings, the STRAP (Stratification of biologic therapies for RA by Pathobiology) study was initiated to further investigate the efficacy of tailored treatments [33]. In this study, patients with rheumatoid arthritis who had shown an inadequate response to csDMARDs were stratified into B cell‐rich and B cell‐poor groups using the same methodology as previously described. The effectiveness of rituximab, tocilizumab, and etanercept was then compared. Regrettably, within the B cell‐poor group, the efficacy was comparable between the rituximab group (59%) and the combined etanercept and tocilizumab group (60%). This indicates that even when patients were stratified using synovial biopsy and transcriptome analysis, these methods did not provide a definitive predictor of treatment response.

These two trials illustrate that while synovial tissue analysis holds potential for advancing precision medicine, the substantial diversity in the underlying pathogenesis of rheumatoid arthritis implies that the complexity of the condition cannot be adequately addressed by a simple dichotomy between patients with a rich B cell signature and those with a poor one. Moreover, when evaluating the effectiveness of targeting B cells versus cytokines, the stratification based on the presence of B cells may have been inappropriate. This is due to the broader impact of inflammatory cytokines, which affect a wide array of cells across various inflammatory pathways. Additionally, it is suggested that using more objective measures, such as X‐ray imaging to assess joint destruction, might provide a more reproducible method for predicting treatment effects compared to using clinical indices like CDAI or ACR20.

## 4 Stratification and Diversity of Peripheral Blood Immune Phenotypes in Patients With Rheumatoid Arthritis

As outlined above, transcriptome analysis utilizing next‐generation sequencers offers high‐resolution insights, allowing for the ex vivo evaluation of patient samples [7, 8]. In contrast, flow cytometry is acknowledged as a more traditional ex vivo method [34]. One key point is that RNA expression does not always correlate directly with protein expression. Flow cytometry excels in detecting subsets, differentiation stages, and activation states in living cells, and it also facilitates the sorting of these cells if required. Additionally, when considering its application in clinical settings, it is important to note that transcriptome analysis can be time‐consuming, and currently, no quicker alternative exists.

**FIGURE 3:**
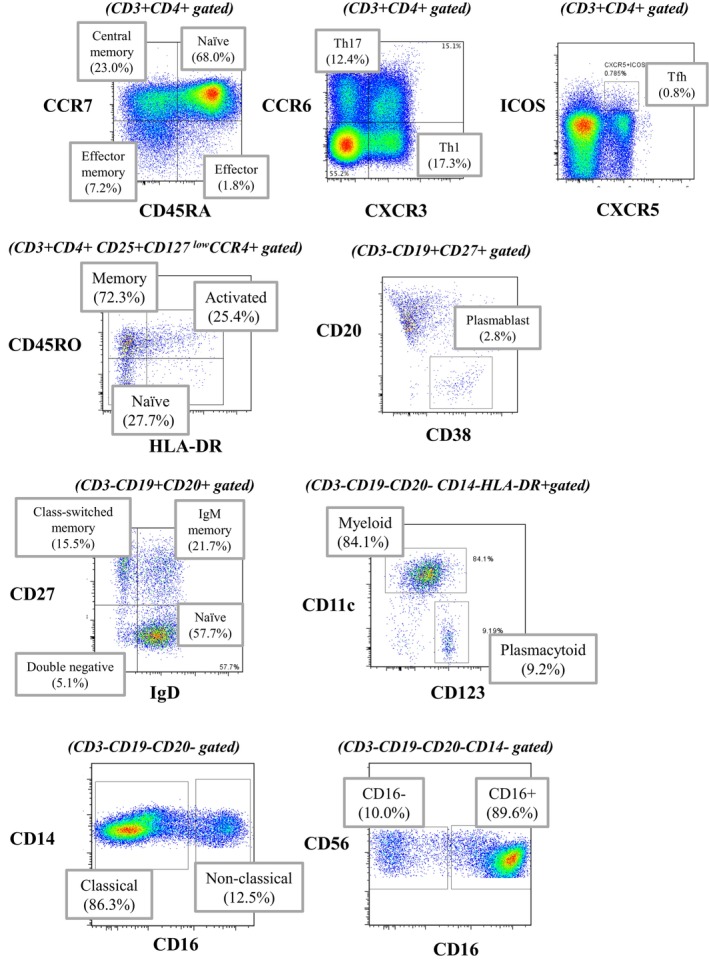
The gating strategy for immunophenotyping using flow cytometry. Different antibody cocktails are utilized to identify various immune cell subsets, including T cells, B cells, dendritic cells, NK cells, and monocytes.

We have previously employed flow cytometry to extract and analyze peripheral blood immune phenotypes across a variety of diseases (Figure 3) [35, 36, 37]. For instance, in the peripheral blood of patients with systemic lupus erythematosus, abnormalities in both B cell and T cell differentiation have been identified. These abnormalities have enabled the stratification of patients into distinct groups, which exhibit varying responses to treatment [38]. In systemic sclerosis, the peripheral blood immune phenotype of approximately half of the patients closely resembled that of healthy controls, suggesting that immune phenotyping of peripheral blood alone could not fully capture the disease's etiology. However, patients exhibiting increased levels of follicular T cells in peripheral blood demonstrate accelerated progression of microvasculopathy [39]. These results suggest that the peripheral blood may relatively reflect the immunological activity occurring at the lesion sites. We thus endeavored to stratify patients based on the peripheral blood immune phenotype from over 700 b/tsDMARDs‐naïve rheumatoid arthritis patients [40]. This study demonstrated that patients with rheumatoid arthritis could be categorized into five distinct groups, referred to as PCAPs (peripheral cell type abundance phenotypes) (Figure 2). Namely, PCAP‐LD (little difference) closely mirrors the immune phenotype of healthy individuals. In contrast, PCAP‐SD (slight difference) shows only minor differences from healthy phenotypes, with notable decreases in activated Tc1 cells and other immune cells. PCAP‐TB (T cell and B cell activation) is characterized by an increase in both CD4^+^ and CD8^+^ T cells, as well as plasmablasts. PCAP‐T4 (TEMRA CD4) displays a significant rise in CD4^+^ TEMRA cells, while PCAP‐T4T1 (TEMRA CD4 and Th1) demonstrates an elevation in both TEMRA and Th1 cells. In terms of clinical background, the PCAP‐TB subgroup often exhibited relatively high disease activity, while the patient backgrounds in the other subgroups appeared similar. This observation suggests that analyzing the peripheral blood immune phenotype can reveal variations in the disease state that are distinct from clinical diversity. Crucially, the effectiveness of each molecular targeted therapy varied across these subgroups. For instance, JAK inhibitors proved effective in the PCAP‐SD subgroup, while both JAK and IL‐6 inhibitors showed efficacy in the PCAP‐LD subgroup. In PCAP‐T4, TNF inhibitors and JAK inhibitors were beneficial, and in PCAP‐T4T1, both CTLA4‐Ig and JAK inhibitors demonstrated effectiveness. On the other hand, in the PCAP‐TB subgroup, the clinical remission rate after 6 months was less than 30% for all drugs, highlighting the challenges in treating this particular phenotype. These findings suggest that by extracting and analyzing immune phenotypes from peripheral blood, it may be possible to optimize treatment selection.

## 5 Requirements for Achieving Precision Medicine in Rheumatoid Arthritis

The EULAR recommendations for the treatment of rheumatoid arthritis are employed globally and have proven effective in bringing patients with a variety of clinical symptoms and conditions into clinical remission. However, achieving optimal treatment outcomes for all patients is challenging with this uniform treatment strategy alone. In contrast, precision medicine aims to maximize treatment efficacy and minimize side effects, potentially leading to dramatic improvements in treatment outcomes.

Recent advancements in science have enhanced our understanding of the pathogenesis of rheumatoid arthritis. Particularly, improvements in immunophenotyping technology have refined the accuracy of ex vivo evaluations using patient samples, facilitating a deeper understanding of patient diversity. Additionally, there is ongoing research into the genetic predispositions associated with rheumatoid arthritis. Approaches that integrate research on genetic polymorphisms with immunoprofiling are paving the way for precision medicine. This combined approach could lead to highly personalized treatments that are tailored to the genetic and immunological profiles of individual patients. However, in reality, numerous challenges persist in achieving precision medicine for rheumatoid arthritis. Currently, it is difficult to develop treatment strategies that comprehensively take into account the immunological background of individual patients. For example, immunophenotyping and genetic analysis are only feasible at a limited number of specialized facilities, and these procedures are associated with high costs. Additionally, the time required for analysis presents further obstacles to their routine use in everyday clinical practice. Furthermore, even if these hurdles can be overcome, proving the effectiveness of precision medicine approaches in rheumatoid arthritis would require large‐scale clinical trials. Conducting these trials demands significant resources and effort. To lay the groundwork for precision medicine, it is crucial to collect and analyze data not only in research environments but also within clinical settings. Additionally, there is a pressing need to train and foster physicians who specialize in translational medicine.

## 6 Conclusion

Achieving precision medicine in the treatment of autoimmune diseases, such as rheumatoid arthritis, is considered a “moonshot” goal. Despite the difficulties, it represents one of the most crucial endeavors in contemporary medical science, aiming to significantly enhance patient outcomes and overall quality of life. In conclusion, this review has underscored the diversity in rheumatoid arthritis that is often overlooked. This disease is influenced by both genetic predispositions and environmental factors, contributing to its complex nature. The future challenge lies in implementing treatments that are tailored to the specific pathology and molecular foundations of each individual patient.

## Author Contributions

Satoshi Kubo contributed to the writing of the manuscript. Yoshiya Tanaka contributed to the overall review. All authors read and approved the final manuscript.

## Conflicts of Interest

Satoshi Kubo has received speaking fees from Eli Lilly, Bristol‐Myers, GlaxoSmithKline, Abbvie, and also research grants from Daiichi‐Sankyo, Abbvie, Boehringer Ingelheim, and Astellas. Yoshiya Tanaka has received consulting fees, speaking fees, and/or honoraria from Abbvie, Daiichi‐Sankyo, Chugai, Takeda, Mitsubishi‐Tanabe, Bristol‐Myers, Astellas, Eisai, Janssen, Pfizer, Asahi‐Kasei, Eli Lilly, GlaxoSmithKline, UCB, Teijin, MSD, and Santen, and also research grants from Mitsubishi‐Tanabe, Takeda, Chugai, Astellas, Eisai, Taisho‐Toyama, Kyowa‐Kirin, Abbvie, and Bristol‐Myers.

## Data Availability

Data sharing not applicable—no new data generated, or the article describes entirely theoretical research.
